# Machine Learning Models for Parkinson Disease: Systematic Review

**DOI:** 10.2196/50117

**Published:** 2024-05-17

**Authors:** Thasina Tabashum, Robert Cooper Snyder, Megan K O'Brien, Mark V Albert

**Affiliations:** 1 Department of Computer Science and Engineering, University of North Texas, Denton, TX, United States; 2 Technology and Innovation Hub, Shirley Ryan AbilityLab, Chicago, IL, United States; 3 Department of Physical Medicine & Rehabilitation, Northwestern University, Chicago, IL, United States; 4 Department of Biomedical Engineering, University of North Texas, Denton, TX, United States

**Keywords:** Parkinson disease, machine learning, systematic review, deep learning, clinical adoption, validation techniques, PRISMA, Preferred Reporting Items for Systematic Reviews and Meta-Analyses

## Abstract

**Background:**

With the increasing availability of data, computing resources, and easier-to-use software libraries, machine learning (ML) is increasingly used in disease detection and prediction, including for Parkinson disease (PD). Despite the large number of studies published every year, very few ML systems have been adopted for real-world use. In particular, a lack of external validity may result in poor performance of these systems in clinical practice. Additional methodological issues in ML design and reporting can also hinder clinical adoption, even for applications that would benefit from such data-driven systems.

**Objective:**

To sample the current ML practices in PD applications, we conducted a systematic review of studies published in 2020 and 2021 that used ML models to diagnose PD or track PD progression.

**Methods:**

We conducted a systematic literature review in accordance with PRISMA (Preferred Reporting Items for Systematic Reviews and Meta-Analyses) guidelines in PubMed between January 2020 and April 2021, using the following exact string: “Parkinson’s” AND (“ML” OR “prediction” OR “classification” OR “detection” or “artificial intelligence” OR “AI”). The search resulted in 1085 publications. After a search query and review, we found 113 publications that used ML for the classification or regression-based prediction of PD or PD-related symptoms.

**Results:**

Only 65.5% (74/113) of studies used a holdout test set to avoid potentially inflated accuracies, and approximately half (25/46, 54%) of the studies without a holdout test set did not state this as a potential concern. Surprisingly, 38.9% (44/113) of studies did not report on how or if models were tuned, and an additional 27.4% (31/113) used ad hoc model tuning, which is generally frowned upon in ML model optimization. Only 15% (17/113) of studies performed direct comparisons of results with other models, severely limiting the interpretation of results.

**Conclusions:**

This review highlights the notable limitations of current ML systems and techniques that may contribute to a gap between reported performance in research and the real-life applicability of ML models aiming to detect and predict diseases such as PD.

## Introduction

Parkinson disease (PD) is a progressive neurodegenerative disease that results in a loss of motor function with muscle weakness, tremors, and rigidity. Secondary symptoms include speech difficulties, sleep disorders, and cognitive changes. Research suggests that pathophysiological symptoms can be used to detect PD before the onset of the motor features [1]. For these reasons, multiple clinical assessments and analyses are required to diagnose PD and allow for early detection. However, clinical diagnosis of PD is an error-prone process [2]. A UK autopsy study found that the misdiagnosis rate of PD is 24% [3]. Early detection is especially important for PD since early neuroprotective treatment slows down the progression of the disease and lessens the symptoms, which improves the patient’s quality of life [4]. From diagnosis to treatment, each case of PD is unique [5,6]. Precision medicine using machine learning (ML) has the potential to better use the varied data of individuals. Therefore, ML-based solutions can play an important role in PD diagnosis [7,8].

Here, ML refers to the branch of artificial intelligence that uses computational methods to perform a specific task without being explicitly programmed, by learning from previous examples of data and making predictions about new data [9]. ML includes a broad range of standard learning algorithms, such as decision trees, support vector machines, and linear or logistic regression, as well as the subfield of deep learning that uses sophisticated, biologically inspired learning algorithms called neural networks. Generally, supervised algorithms learn from labeled data (eg, classification or regression), whereas unsupervised algorithms learn from hidden patterns in the unlabeled data (eg, clustering).

In the medical field, ML is becoming an increasingly central technique. For example, ML-based prediction models are being developed to detect early signs of diseases, improve decision-making processes, and track rehabilitation efficacy. Fueled by advances in data-recording technology, the increasing availability of patient data, and more accessible databases and code libraries, these models can generate more accurate insights about patients from large, existing health data sets. Contreras and Vehi [10] showed that within a decade, the number of articles proposing artificial intelligence models in diabetes research grew by 500%. Despite the large number of promising studies reported in the literature, the adoption of ML models in real-life clinical practice is low [11]. A wide range of ML models have been proposed for the automatic detection of PD [12]. Searching with only 1 query related to ML and PD results in over 1000 publications in 1 year alone. Despite the rising popularity of ML in PD research, models are rarely deployed in the field due to their irreproducibility and are limited for research purposes [13]. Although there may be many explanations, one possibility is a disconnect between the models developed in research and real-life implementation.

In contrast to previous systematic reviews that primarily explored data types and model variations, the emphasis of this review lies in the critical context of model validation approaches to provide a comprehensive understanding of the strengths and limitations of ML models in the PD field. Previous reviews emphasized data types; for instance, Ramdhani et al [14] reviewed sensor-based ML algorithms for PD predictions, and Mei et al [15] provided a comprehensive overview of outcomes associated with the type and source of data for 209 studies that applied ML models for PD diagnosis. Mei et al [15] also noted concerns about insufficient descriptions of methods, results, and validation techniques. We focused on the critical evaluation of validation techniques that are instrumental for the clinical integration of ML.

In this review, we examined a cross-section of recent ML prediction models related to PD detection and progression. Our goal was to summarize the different ML practices in PD research and identify areas for improvement related to model design, training, validation, tuning, and evaluation. Implementing best ML practices would help researchers develop PD prediction models that are more reproducible and generalizable, which in turn would improve their impact on the entire landscape of patient care and outcomes.

## Methods

### Search Strategy

We conducted a systematic literature review in accordance with PRISMA (Preferred Reporting Items for Systematic Reviews and Meta-Analyses; Checklist 1) guidelines in PubMed between January 2020 and April 2021, using the following exact string: “Parkinson’s” AND (“ML” OR “prediction” OR “classification” OR “detection” or “artificial intelligence” OR “AI”). The search resulted in 1085 publications.

### Inclusion and Exclusion

Inclusion criteria were studies (1) on ML applied for predicting PD, PD subscores or PD severity, and PD symptoms; (2) published between January 2020 and April 2021; (3) written in English; and (4) with an available title and abstract.

### Questionnaire Design

We designed a customized questionnaire to easily parse the literature and extract characteristics of the different ML approaches. Textbox 1 summarizes the model details extracted from the questionnaire, and the exact questionnaire is provided in Multimedia Appendix 1. This questionnaire was not intended to extract exhaustive details about these models, but rather to target specific concepts that seem to be inconsistently reported in the PD modeling literature. Our rationale for each question, and how they were designed specifically for PD, is provided below.

PD is a progressive neurological disorder, and symptoms can vary widely for each individual. To categorize PD progression and assess patient status, clinicians use standardized metrics such as the Unified Parkinson’s Disease Rating Scale [16] and Hoehn and Yahr (H&Y) scores [17]. The first question is related to clearly defining the research objectives or target outcomes of a particular study. The challenge of classifying PD versus non-PD may depend on symptom severity, which can be more readily assessed when severity metrics are available. In certain stages of PD, symptoms can be controlled or lessened through careful medication regimens, such as levodopa. This medication’s *on* and *off* periods are essential components for clinicians and researchers to consider. *On* and *off* episodes can create a substantially different effect on symptoms [18,19], and these symptoms are being used in ML algorithms to classify or assess PD. For example, Jahanshahi et al [20] investigated the levodopa medication’s effect on PD probabilistic classification learning and demonstrated that learning is associated with the patient with PD being in an *on* or *off* state. Warmerdam et al [21] showed that the patient’s state relative to dopaminergic medication correlated with the arm-swing task during PD walking. PD characteristics are important while researching PD, and the application of the models might play different roles depending on the data. As a result, the questions regarding the severity and medication state of patients can play a crucial role. In addition, class imbalance, cross-validation techniques, and hyperparameter tuning are critical concepts in ML. Class imbalance can lead to biased models or misinterpretation of results. Cross-validation and hyperparameter tuning allow systematic exploring of models and are essential for assessing models’ generalization performance. Lastly, comparing model performance to benchmark data can be valuable for research goals, but this process is not always applicable or possible.

Textbox 1. **Model details obtained during data extraction (n=113).**

1. What have the authors classified using machine learning?
2. Was there any information about the participants being on or off medication prior to the experiment?
3. Of the study participants, how many were (1) individuals with Parkinson disease, (2) controls, and (3) individuals with other diseases?
4. Did the study mention the distribution of the Unified Parkinson’s Disease Rating Scale and Hoehn and Yahr scores?
5. What class imbalance mitigation techniques did the authors perform?
6. How did the authors split or cross-validate the data set while training the model? If cross-validation was applied, which particular strategies were applied?
7. If applicable, have the authors made the reader aware of the potential overinflated performance results (eg, the model overfitting the training data)? If so, how?
8. How was the hyperparameter tuning done?
9. Did the authors analyze and discuss the models’ errors or misclassifications?
10. How did they compare their model to other modeling approaches by themselves or other authors, directly or indirectly?
11. Did the authors use multiple evaluation metrics to measure the performance of the model(s)?

### Data Extraction

Two authors assessed the inclusion criteria of 1085 studies based on the title and abstract. During the initial manual screening of the title and abstract, 155 studies that met the initial inclusion criteria were identified. A total of 42 studies were excluded after assessing the full text for eligibility. These authors also extracted data from the studies using the questionnaire described above. Ultimately, 113 studies and the corresponding questionnaire responses were rechecked independently by both reviewers, and disagreements were resolved through discussion to reach a consensus. Questionnaire data from each study are provided in in Multimedia Appendix 2.

For the multiple-choice and checkbox questions (ie, questions 1, 7, 8, 9, 10, 11, 13, 14, and 15), we counted the number of times each response occurred in the results.

## Results

First, we provide a general overview of the study characteristics in each publication. Then, we examine specific results evaluating the ML modeling practices using the following categories: PD characteristics, class imbalance, data set splitting, overfitting, hyperparameter tuning, and model comparisons.

### General Overview of Studies

#### Methods Applied

The most prevalent ML classification algorithms were support vector machines (53/113, 46.9%), boosting ensemble learning (48/113, 42.5%; eg, gradient boosting, extreme gradient boosting, and random forest), naive Bayes (4/113, 3.5%), decision tree (13/113, 11.5%), and *k*-nearest neighbor (22/113, 19.5%). In regression models, the most prevalent methods included multiple linear or logistic regression (32/113, 28.3%), regression trees, *k*-means clustering, and Bayesian regression (3/113, 2.6%). Deep learning methods included convolutional neural networks (10/113, 8.8%), variants of recurrent neural networks (4/113, 3.5%; eg, long short-term memory [LSTM] and bidirectional-LSTM), and fully connected neural networks (22/113, 19.5%).

#### Data Modalities and Sources

More than half of the studies (65/113, 57.5%) used data collected by the authors, whereas 38.9% (44/113) used a public data set and 3.6% (4/113) used a mixture of public and private data sets. The most common data modalities were magnetic resonance imaging, single-photon emission computerized tomography imaging, voice recordings or features, gait movements, handwriting movements, surveys, and cerebrospinal fluid features.

### ML Modeling Practices

#### PD Prediction Target

We categorized the studies based on 5 ML outcomes for PD models: *PD versus non-PD classification*, *PD severity prediction*, *PD versus non-PD versus other diseases classification*, *PD symptoms quantification*, and *PD progression prediction*. A total of 10 studies fell into more than 1 category; among them, 8 (80%) studies examined both *PD versus non-PD classification* and *PD severity regression*, and 2 (20%) studies examined *PD versus non-PD classification* and *PD symptoms quantification*.

1. *PD versus non-PD classification* (59/113, 52.2%): studies that proposed ML methods to distinguish between individuals with PD from controls without PD
2. *PD severity prediction* (30/113, 26.5%): studies that proposed ML methods to predict the stages of Unified Parkinson’s Disease Rating Scale scores or H&Y scores of PD
3. *PD versus non-PD versus other diseases classification* (24/113, 21.2%): studies that proposed ML methods to distinguish between PD, non-PD, and other diseases (eg, Alzheimer disease)
4. *PD symptoms quantification* (9/113, 8%): studies that proposed ML methods to distinguish between PD symptoms (eg, tremor and bradykinesia) from no symptoms or non-PD symptoms
5. *PD progression prediction* (1/113, 0.9%): studies that proposed ML methods to predict PD progression

*PD versus non-PD classification* and *PD versus non-PD versus other diseases classification* have target settings that are binary variable predictions, as these targets are mostly for predicting the presence or absence of PD. *PD severity prediction* can be categorical (multilabel classification) or continuous (regression), such as predicting the H&Y score. *PD symptoms quantification* can also be categorical, such as predicting the presence of resting tremors, rigidity, and bradykinesia, or continuous, such as predicting the degree of tremor intensity. *PD progression prediction* measures the changes in overall disease severity at multiple time points. We found that most studies (107/113, 94.6%) indicated PD severity. However, fewer than half (53/113, 46.9%) of the studies reported the patient medication status directly, with 38.9% (44/113) using public data sets.

#### Class Imbalance

Class imbalance occurs when 1 training class contains significantly fewer samples than another class. In this case, the learners tend to focus on the better performance of the majority group, making it difficult to interpret the evaluation metrics, such as accuracy, for groups with less representation. Prediction models can be significantly affected by the imbalance problem. ML models can be highly unstable with different imbalance ratios [22]. On predicting *PD versus non-PD classification*, performance can suffer significantly from an imbalanced data set and generate impaired results [23]. Class imbalance can impact model external validity, and either mitigating or at least reporting the potential concerns in the interpretability of outcomes due to imbalances would help the reader interpret the model’s power for predicting each class.

**Figure 1.**
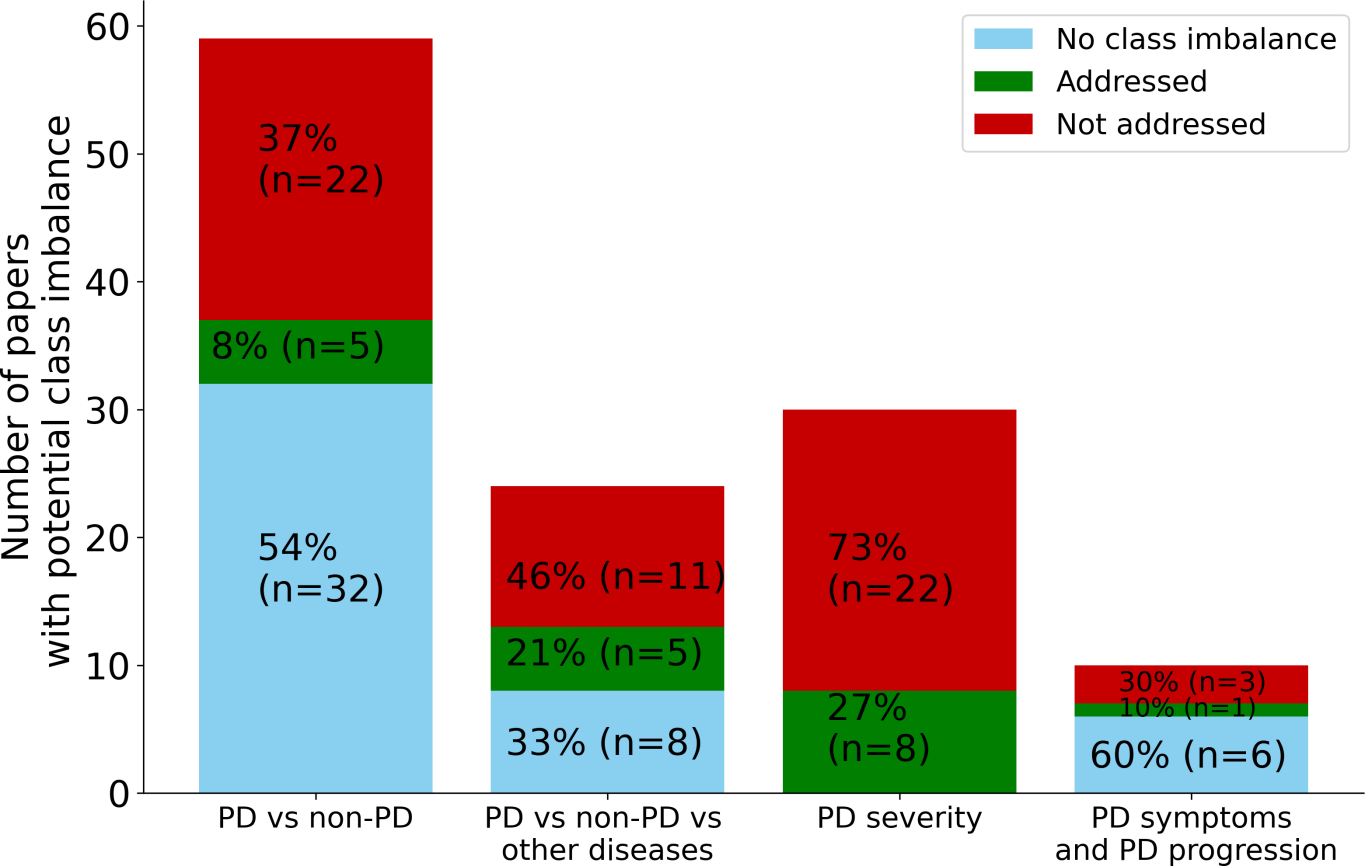
Number of studies with more than 30% class imbalance and the percentage of studies that applied the class imbalance strategies, separated by PD prediction target. In the *PD versus non-PD classification*, *PD versus non-PD versus other diseases classification*, *PD severity prediction*, and *PD symptoms quantification and progression prediction* categories, 46% (27/59), 67% (16/24), 100% (30/30), and 40% (4/10) had class imbalance, but only 8% (5/59), 21% (8/30), 27% (8/30), and 10% (1/10) applied mitigation strategies, respectively. PD: Parkinson disease.

There are multiple ways to handle a class imbalance in the training phase, such as using resampling techniques or weighted evaluation metrics. Resampling creates a more balanced training data set, such as by oversampling the minority class or undersampling the majority class [24,25]. Moreover, there are alternative evaluation metrics, for example, balanced accuracy and *F*-measure, but these improvements on the standard evaluation metrics are also affected by class imbalance [26]. We observed that among the studies that attempted to mitigate class imbalance, many of them adopted under- or oversampling methods and then applied class weights to the evaluation metrics. Other techniques were data augmentation and grouping data to use the same ratio of minority and majority classes. In the case of extreme class imbalance, Megahed et al [27] were not able to mitigate overfitting. Overall, there is no perfect solution to tackle this critical issue in ML; however, recognizing that the problem exists and investigating appropriate mitigation strategies should be standard practice. Our results found at least moderate class imbalance in more than two-thirds (77/113, 68.1%) of the studies, and only 18% (5/27), 31% (5/16), 27% (8/30), and 25% (1/4) of studies for the *PD versus non-PD classification*, *PD versus non-PD versus other diseases classification*, *PD severity prediction*, and *PD symptoms quantification and progression prediction* target categories applied strategies to mitigate the effects of class imbalance, respectively. In Figure 1, we illustrate the number of studies with more than 30% class imbalance and how many of them applied imbalance mitigation strategies.

In some cases, authors applied class imbalance strategies but found no significant improvement in their model performance. Reporting these cases still provides valuable perspectives. For instance, van den Goorbergh et al [28] illustrated that correcting for imbalance resulted in the model exhibiting strong miscalibration and did not improve the model’s capability to distinguish between patients and controls. A total of 4 studies compared results when using imbalanced data compared to imbalance-mitigated data. Details of these studies are provided in Table 1.

**Table 1.** Comparison between imbalanced data versus imbalance mitigation strategies.

Studies	Participant distribution	Techniques	Conclusion
Moon et al [29]	524 patients with PD^a^ and 43 patients with essential tremor	SMOTE^b^	*F*_1_-score improved
Veeraragavan et al [30]	93 patients with idiopathic PD and 73 controls; 10 patients with H&Y^c^ 3; 28 patients with H&Y 2.5; and 55 patients with H&Y 2	SMOTE	Test accuracy improved
Falchetti et al [31]	388 patients with idiopathic PD and 323 controls	Oversampling Undersampling Combination of oversampling and undersampling	Without any sampling, the combination of oversampling and undersampling methods is comparable
Jeancolas et al [32]	115 patients with PD and 152 controls	Data augmentation	Performed better for free speech task No consistent improvement in the sentence repetition task

a PD: Parkinson disease.

b SMOTE: synthetic minority oversampling technique.

c H&Y: Hoehn and Yahr.

#### Data Set Splitting

It is universally acknowledged that ML models can perform arbitrarily well on data that were used to create the model—that is, the training data set. This is why standard procedure in training models uses separate data sets to try different model variations and select the better variants. The confusion that sometimes occurs is when these separate data sets are used to select from a large number of model variants (validation set) or only used for the evaluation of selected variants (test set). The distinction in these 2 use cases of separate data is sometimes not clear and depends on the number of model variants tested. Critically, with modern ML practice, many model variants are often tested on provided data, which readily leads to overfitting on both the original training data and validation set used for evaluation. A separate holdout test set would be needed to properly evaluate model performance [33]. A single split can be error prone in estimating performance [34]. It is critical to have a holdout test set to provide better performance estimation. Additionally, cross-validation is a technique largely used to estimate and compare model performance or to optimize the hyperparameters [35]. Cross-validation divides the data into folds and iterates on these folds to test and train the models using different partitions of the data set. We found that 78.8% (89/113) of the studies used cross-validation; however, 5.3% (6/113) of the studies either did not mention the details of the validation procedure or did not do any splitting. A total of 9.7% (11/113) of the studies split the data set into only 2 sets, but it was not clear if the separate set was a validation set or a test set. Only 19.5% (22/113) of the studies applied cross-validation without a holdout test set (Table 2 and Figure S1 in Multimedia Appendix 3).

**Table 2.** Distribution of studies according to data set splitting techniques.

Data set splitting techniques	Studies (n=113), n (%)
Not mentioned	6 (5.3)
Split into 2 sets (training, test, or validation sets)	11 (9.7)
Only cross-validation	22 (19.5)
Split into 3 sets	7 (6.2)
Cross-validation and holdout test set	67 (59.3)

#### Cross-Validation

There are multiple types of cross-validation techniques. In *k*-fold cross-validation, the data set is divided into *k* equal folds randomly, and the model is trained and evaluated *k* times. Each time, the model is trained using *k*–1 folds and evaluated in the remaining fold. When the observations are independent and identically distributed, *k*-fold cross-validation works well. When the data are not identically distributed, *k*-fold cross-validation makes the model prone to overfitting and not generalize well [36]. For instance, multiple data samples from the same patient should generally not be present in both training and testing data sets. Subject-wise cross-validation separates folds according to the subject. Although Saeb et al [37] concluded that subject-wise methods are more clinically relevant compared to record-wise methods, Little et al [38] argued that subject-wise methods might not be the best in all use cases. However, Westerhuis et al [39] demonstrated that cross-validation can be overoptimistic and suggested that it is good practice to include a separate test set at the end to properly evaluate a model. To reduce bias in model evaluation, nested cross-validation is another technique that involves 2 cross-validation loops [40]. The outer loop generates *k*-folds and iterates through them, so each fold is eventually used as a holdout test fold for a model developed using the remaining data. The inner loop uses a similar *k*-fold procedure to create a holdout validation fold that is used to select the best model during model tuning. Nested cross-validation is a more robust way to evaluate models than *k*-fold cross-validation alone, since using all available data to select the model architecture can lead to biased, overfitted results [40]. However, nested cross-validation is more computationally intensive, and these models can be difficult to interpret or implement (since they actually result in *k*-best models, so performance is usually averaged over all *k*-best models). In our analysis, we found that the most common cross-validation technique is *k*-fold cross-validation (68/113, 60.2%), whereas only 4.4% (5/113) of the studies adopted nested cross-validation (Table 3 and Figure S2 in Multimedia Appendix 3). Of the 113 studies, 20 (17.7%) adopted 2 types of cross-validation techniques, and 5 (4.4%) adopted 3 types of techniques.

**Table 3.** Distribution of studies that adopted cross-validation techniques.

Cross-validation techniques	Studies (n=113), n (%)
*k*-fold cross-validation	68 (60.2)
Leave-p-out cross-validation	25 (22.1)
Stratified or subject-wise cross-validation	21 (18.6)
Nested cross-validation	5 (4.4)
No cross-validation	24 (21.2)

#### Overfitting

We selected publications that did not evaluate their models with a holdout test set and then we analyzed if they mentioned that the proposed models could possibly be overfitting. Models can be overfitted for multiple reasons, such as an imbalanced data set or the lack of proper model selection and validation technique. Even with cross-validation, if a separate holdout set is not used, then the results can be inflated. Rao et al [41] demonstrated that leave-one-out cross-validation can achieve 100% sensitivity, but performance on a holdout test set can be significantly lower. Cross-validation alone is not sufficient model validation when the dimensionality of the data is high [41]. However, there are multiple ways to address or prevent overfitting, such as the examples provided by Ying [42]. Making the reader aware of overfitting concerns in the interpretability of results should be standard practice. Therefore, we searched to see if the authors mentioned that their model can suffer from overfitting. For this analysis, we excluded studies that applied the cross-validation technique with a holdout test set. We found that just over 54% (25/46) of the studies that likely suffer from overfitting did not mention it as a concern. Although 45% (21/46) of studies mentioned overfitting as a potential limitation, many of them did not have any detailed discussion about this.

#### Hyperparameters

While training a model, hyperparameters are selected to define the architecture of the model. These hyperparameters are often tuned so that the model gives the best performance. A common method of finding the best hyperparameters is by defining a range of parameters to test, then applying a grid search or random search on the fixed search space, and finally selecting parameters to minimize the model error [43]. These methods can be extremely computationally expensive and time-consuming depending on data complexity and available computation power [44]. Regardless of the method applied, it is considered good practice to make clear statements about the tuning process of hyperparameters to improve reproducibility [45]. This practice ensures parameters are properly selected and models are ready for direct comparison. Our results demonstrated that 38.9% (44/113) of studies did not report on hyperparameter tuning (Table 4 and Figure S3 in Multimedia Appendix 3). Of these, 2 adopted least absolute shrinkage and selection operator logistic regression, and 3 used a variant of logistic regression or linear regression, which typically have few or no hyperparameters to adjust.

**Table 4.** Distribution of studies according to hyperparameter tuning methods.

Hyperparameter tuning methods	Studies (n=113), n (%)
Not reported	44 (38.9)
Ad hoc	31 (27.4)
Random search	1 (0.9)
Grid search	27 (23.9)
Others	10 (8.8)

For many other models, there are inherently only a few hyperparameters that are usually adjusted; for instance, the major hyperparameter for the neighbor model is the number of neighbors, *k*. On the other hand, more complex models such as convolutional neural networks and LSTM require thorough tuning to achieve meaningful performance. Regardless of the number of hyperparameters in a model, proper tuning would likely still contribute to achieving optimal performance. The choice of hyperparameters will impact model generalization, so it is worthwhile to examine changes in performance with different settings [46].

#### Model Comparison

In research domains that require complex deep learning models to achieve state-of-the-art performance, such as computer vision and natural language processing, it has become a regular practice to compare models with numeric benchmark data sets to contextualize their proposed model and provide insight into the model’s relative performance to peers. Although such rigorous benchmarking and comparison is not possible given the heterogeneous data sets in PD research, it is important to contextualize a model’s performance relative to other models, strategies, and data sets. We found that 66.4% (75/113) of studies compared results from multiple alternative models in their work, and 15% (17/113) of studies compared their results with previously published models. However, 18.6% (21/113) of studies only reported their single model performance and made no comparison to any other models or benchmarks (Table 5 and Figure S4 in Multimedia Appendix 3).

**Table 5.** Distribution of studies according to model comparison methods; 18.6% (21/113) of studies did not compare their model results to any alternative models or previously published models or benchmarks.

Model comparison methods	Studies (n=113), n (%)
Compared with their own multiple models	75 (66.4)
Compared with previous models or benchmarks	4 (3.5)
Compared with previous models and their own multiple models	13 (11.5)
No comparisons	21 (18.6)

## Discussion

### Principal Findings

In summary, we have comprehensively reviewed the general practices of ML research applied to PD in a recent cross-section of publications. We have identified several important areas of improvement for model building to reduce the disparity between in-the-lab research and real-world clinical applications. Standardizing the model reporting techniques and implementing best ML practices would increase the acceptability and reliability of these models to improve patient evaluation and care [47].

For the interoperability and usability of the models, clinicians need detailed information about the patients included in the model’s training data, such as their medication state and PD progression stage. This information determines the predictive validity of a model to new patients and settings. We found that 94.7% (107/113) of the studies explained the PD severity of their patients, whereas only 46.9% (53/113) of studies reported the medication state of the patients. To incorporate data-driven algorithms in real life, the description of medication is significantly relevant to PD [48,49]. The overall representation of demographic samples in the training set should be accounted for as well. Our results show that 68.1% (77/113) of the studies had a class imbalance greater than 30% difference in their data set, and less than one-third (from 5/27, 18% to 5/16, 31%) of the studies addressed imbalance as a potential issue or considered its impact on the model results.

Another major finding is the lack of a standard reporting framework for a model’s hyperparameter search and tuning. Hyperparameter tuning has a major impact on the model configuration and, by extension, its performance [50]. For example, Wong et al [51] demonstrated that a model using tuned (grid-searched) hyperparameters outperformed a model using default hyperparameters. Addressing hyperparameters is also essential for reproducibility, including a report on the final model configuration and how the authors made the decision. Although this is a considerably important aspect of ML model reporting, our study showed that 44 (38.9%) of the 113 studies did not report the hyperparameter tuning approach. Of these, 5 studies adopted logistic regression or linear regression. Traditional regression models are not expected to undergo significant hyperparameter tuning; however, variants that involve hyperparameters would likely still benefit from tuning. Consistent reporting of hyperparameter tuning practices will enhance the robustness and reliability of these models.

Moreover, to provide context to the results of model performance, comparisons of different models or with previously published models give a general idea of the quality of the proposed models. We found that 18.6% (21/113) of the studies only reported their proposed models; on the contrary, the reporting standard of proposed models in the computer vision and natural language processing fields is extensive. For instance, Wang et al [52] and Liu et al [53] proposed methods for visual recognition, and they reported large-scale experiment results with different data sets and compared their results with more than 10 previously proposed methods. Similarly, in natural language processing, to propose a task such as emotion cause extraction, Xia and Ding [54] compared around 8 methods with different evaluation metrics. These are a few cases to demonstrate that such comparisons are widely executed in the computer vision and natural language processing communities to propose a method. This systematic practice of comparison with previously published approaches results in reproducibility. Unfortunately, we found that only 15% (17/113) of the studies compared with previously proposed methods. However, in the medical field, due to the challenges of data availability, proper comparisons might not be possible.

There are several factors in ML and deep learning research that can create misleading results. One major factor is proper model validation, particularly in how the training and test data are separated. We found that 5.3% (6/113) of studies either did not provide the details about data set splitting or did not do any splitting, and 15.9% (18/113) of studies performed static training, validation, and test set separation, which provides limited stability of scores. Cross-validation is a more stable validation method conducted while training the model and reduces the risk of overfitting [55]. The majority (89/113, 78.8%) of studies adopted some form of cross-validation, and the most common cross-validation technique adopted was *k*-fold (68/113, 60.2%). Nevertheless, the use case of different validation techniques depends on the data set and is problem specific. As powerful as cross-validation is in creating reliable models, applying simple cross-validation does not guarantee that the model is not overfitted [41]. For the studies that did not evaluate their results with a holdout test set in a cross-validation manner, we extracted information from their discussion sections. To be precise, we checked if they made their reader aware of how the study results might be overfitting. We found that 46% (21/46) of the studies that are potentially reporting overfitted scores did not mention this concern. The developed models should be reported with their limitations for transparency to allow for further improvement and real-world adoption.

In this systematic review, we sampled 113 recent studies on PD to summarize the standard ML practices and addressed broader concerns on reporting strategies. It is challenging for authors to always implement the best practices considering the practical realities of health care data, including limited sample sizes, noisy data, medical data privacy, etc. However, whenever possible, authors should consider these reporting practices, especially to acknowledge limitations in their data, model design, and performance. This will help to determine reasonable use cases for these models or to identify areas of improvement before they are ready for clinical translation. These considerations can also extend to other health care applications of ML.

### Conclusion

Despite the increasing number of studies, our results demonstrate there are still many opportunities for improvement in reporting and implementing ML for applications in PD detection and progression. Studies should report detailed, standardized patient characteristics; use robust validation techniques to ensure the model’s reliability; and justify choices of evaluation as well as hyperparameters. We found that 75% (58/77) of the studies sampled from 2020 to 2021 did not address class imbalance, and one-third (44/113, 38.9%) of studies did not report hyperparameter tuning. Reporting is the first step to understanding the usability and interpretation of models. By shifting the focus to the critical evaluation of these methods, we aim to improve the reporting and review of ML to strengthen the connection between research and real-world clinical applications. Ideally, the processes can be standardized, and clinical measurements can be leveraged more effectively for prediction models to improve the real-world impact on individuals with PD or other health conditions.

## Supplementary material

10.2196/50117 Multimedia Appendix 1 Customized questionnaire.

10.2196/50117 Multimedia Appendix 2 List of included studies.

10.2196/50117 Multimedia Appendix 3 Graphical representations of data.

10.2196/50117 Checklist 1 PRISMA (Preferred Reporting Items for Systematic Reviews and Meta-Analyses) checklist.

## Abbreviations

H&Y: Hoehn and Yahr
LSTM: long short-term memory
ML: machine learning
PD: Parkinson disease
PRISMA: Preferred Reporting Items for Systematic Reviews and Meta-Analyses
