# Supplementary material for: Machine Learning Models for Parkinson Disease: Systematic Review
Source: JMIR Med Inform. 2024 May 17;12:e50117. doi: 10.2196/50117 (PMC11112052; doi:10.2196/50117)
Supplement: Multimedia Appendix 1 [file medinform-v12-e50117-s001.pdf]

Supplementary Table 1:

Data curation Questions

Questions	Options
1. What have the authors classified using machine learning?	☐ PD vs Non-PD<br>☐ PD, Non-PD and other diseases<br>☐ PD severity (Unified Parkinson Disease Rating Scale (UPDRS) or Hoehn and Yahr (H&R))<br>☐ PD Symptoms (UPDRS scores)
2. How many PD subjects were in this study?	Fill in the blank.
2.1 If there are subcategories of PD, what is the distribution of those categories? For example {"PD-NC": 35, "PD-MCI": 58}' otherwise "Not Mentioned"	Fill in the blank.
3. If reported, what were the mean and standard deviations of the PD subjects' Hoehn and Yahr scores? If not reported, put, "Not Mentioned". If raw data is reported, put "Raw data"	Fill in the blank.
3.1 Copy and paste the distribution data for Hoehn and Yahr scores.	Fill in the blank.
4. If reported, what were the mean and standard deviations of the PD subjects' UPDRS scores? If not reported, put, "Not Mentioned". If raw data is reported, put "Raw data"	Fill in the blank.
4.1 Copy and paste the distribution data for UPDRS scores.	Fill in the blank.
5. How many control subjects are in this study if any? If there was no control subjects just mention "No Control"	Fill in the blank.
6. How many other disease subjects? "If there is no other disease subjects mention "No other disease"	Fill in the blank.
7. Was there any information about the subjects being on or off medication for periods of time prior to the experiment?	○ Not Mentioned<br>○ Medication OFF<br>○ Medication ON<br>○ Mentioned, but the authors did not direct patients to be on or off their medication.<br>○ Both Medication ON and OFF

8. What class imbalance mitigation techniques did the authors perform?	☐ Over/Under Sampling<br>☐ Class weights on evaluation metrics'<br>☐ The authors did not report any class imbalance mitigation strategies<br>☐ Other
9. How did the authors of the paper split/cross-validate the dataset while training the model?	○ The authors did not split the dataset into separate training and test sets and did not use cross-validation (did not separate train & test sets at all).<br>○ The authors performed only cross-validation (train and "test" sets separated through cross-validation only. No use of a hold-out test set for final evaluation.)<br>○ The authors performed cross-validation while training the model, followed by a hold-out test set evaluation.<br>○ The authors split the data into three distinct sets; a training set, a hold-out validation set, and a hold-out test set. (No cross-validation was performed)<br>○ Did not mention anything about data splitting<br>○ Only separated train and test set
10. If cross-validation was applied, which one(s) were applied?	☐ K-fold cross-validation<br>☐ subject-wise cross-validation<br>☐ stratified cross-validation<br>☐ nested cross-validation<br>☐ leave one out cross-validation<br>☐ record-wise cross-validation<br>☐ Did not do any cross-validation
11. Have the authors made the reader aware of the potential of the model(s) having overinflated performance results (e.g. the model overfitting the training data)? If so, how?	○ No, not addressed at all.<br>○ Yes, brief mention of the concept and the term overfitting.<br>○ Yes, in-depth discussion about overfitting and the need for a hold-out test set to mitigate overinflated results.
11.1 Copy and paste the overfitting discussion.	Fill in the blank.
12. Did the authors include a section analyzing the reasons for the models' errors/misclassifications?	○ Yes,<br>○ No
12.1 Copy and paste the error/misclassification analysis.	Fill in the blank.
13. How did they compare their model?	☐ Compared with benchmarks/previous models<br>☐ Compared with their own multiple models<br>☐ Made a comparison after real-life deployment

	☐ Not mentioned (Only reported results of one model)
14. How has the hyperparameter tuning been done?	☐ Ad-Hoc (Researchers' best guess and/or trial and error)<br>☐ Grid Search (Systematic method of trying many options)<br>☐ Random Search (Defining a search space and random sampling those options)<br>☐ Not mentioned (Please double check)
15. Did the authors use multiple evaluation metrics to measure the performance of the model(s)?	○ No, only one metric such as accuracy was reported.<br>○ Yes, multiple metrics were reported.
