# Supplementary material for: Machine Learning Models for Parkinson Disease: Systematic Review
Source: JMIR Med Inform. 2024 May 17;12:e50117. doi: 10.2196/50117 (PMC11112052; doi:10.2196/50117)
Supplement: Multimedia Appendix 2 [file medinform-v12-e50117-s002.pdf]

Supplementary Material 2: S2

1. San-Segundo R, Zhang A, Cebulla A, Panev S, Tabor G, Stebbins K, et al. Parkinson's Disease Tremor Detection in the Wild Using Wearable Accelerometers. Sensors . 2020;20. doi:10.3390/s20205817

2. Zhang J, Li Y, Gao Y, Hu J, Huang B, Rong S, et al. An SBM-based machine learning model for identifying mild cognitive impairment in patients with Parkinson's disease. J Neurol Sci. 2020;418: 117077.

3. Salmanpour MR, Shamsaei M, Saberi A, Klyuzhin IS, Tang J, Sossi V, et al. Machine learning methods for optimal prediction of motor outcome in Parkinson's disease. Phys Med. 2020;69: 233–240.

4. Tsiouris KM, Konitsiotis S, Koutsouris DD, Fotiadis DI. Prognostic factors of Rapid symptoms progression in patients with newly diagnosed parkinson's disease. Artif Intell Med. 2020;103: 101807.

5. Murugappan M, Alshuaib W, Bourisly AK, Khare SK, Sruthi S, Bajaj V. Tunable Q wavelet transform based emotion classification in Parkinson's disease using Electroencephalography. PLoS One. 2020;15: e0242014.

6. Aghanavesi S, Bergquist F, Nyholm D, Senek M, Memedi M. Motion Sensor-Based Assessment of Parkinson's Disease Motor Symptoms During Leg Agility Tests: Results From Levodopa Challenge. IEEE J Biomed Health Inform. 2020;24: 111–119.

7. Chougar L, Faouzi J, Pyatigorskaya N, Yahia-Cherif L, Gaurav R, Biondetti E, et al. Automated Categorization of Parkinsonian Syndromes Using Magnetic Resonance Imaging in a Clinical Setting. Mov Disord. 2021;36: 460–470.

8. Karapinar Senturk Z. Early diagnosis of Parkinson's disease using machine learning algorithms. Med Hypotheses. 2020;138: 109603.

9. Moon S, Song H-J, Sharma VD, Lyons KE, Pahwa R, Akinwuntan AE, et al. Classification of Parkinson's disease and essential tremor based on balance and gait characteristics from wearable motion sensors via machine learning techniques: a data-driven approach. J Neuroeng Rehabil. 2020;17: 125.

10. Aich S, Pradhan PM, Chakraborty S, Kim H-C, Kim H-T, Lee H-G, et al. Design of a Machine Learning-Assisted Wearable Accelerometer-Based Automated System for Studying the Effect of Dopaminergic Medicine on Gait Characteristics of Parkinson's Patients. J Healthc Eng. 2020;2020: 1823268.

11. Cao X, Wang X, Xue C, Zhang S, Huang Q, Liu W. A Radiomics Approach to Predicting Parkinson's Disease by Incorporating Whole-Brain Functional Activity and Gray Matter Structure. Front Neurosci. 2020;14: 751.

12. Ammour A, Aouraghe I, Khaissidi G, Mrabti M, Aboulem G, Belahsen F. A new semi-supervised approach for characterizing the Arabic on-line handwriting of Parkinson's disease patients. Comput Methods Programs Biomed. 2020;183: 104979.

13. De Vos M, Prince J, Buchanan T, FitzGerald JJ, Antoniades CA. Discriminating progressive

supranuclear palsy from Parkinson's disease using wearable technology and machine learning. Gait Posture. 2020;77: 257–263.

14. Ricci M, Di Lazzaro G, Pisani A, Mercuri NB, Giannini F, Saggio G. Assessment of Motor Impairments in Early Untreated Parkinson's Disease Patients: The Wearable Electronics Impact. IEEE J Biomed Health Inform. 2020;24: 120–130.

15. Vaccaro MG, Sarica A, Quattrone A, Chiriaco C, Salsone M, Morelli M, et al. Neuropsychological assessment could distinguish among different clinical phenotypes of progressive supranuclear palsy: A Machine Learning approach. J Neuropsychol. 2021;15: 301–318.

16. Lin C-H, Chiu S-I, Chen T-F, Jang J-SR, Chiu M-J. Classifications of Neurodegenerative Disorders Using a Multiplex Blood Biomarkers-Based Machine Learning Model. Int J Mol Sci. 2020;21. doi:10.3390/ijms21186914

17. Prasuhn J, Heldmann M, Münte TF, Brüggemann N. A machine learning-based classification approach on Parkinson's disease diffusion tensor imaging datasets. Neurol Res Pract. 2020;2: 46.

18. Chen P-H, Lien C-W, Wu W-C, Lee L-S, Shaw J-S. Gait-Based Machine Learning for Classifying Patients with Different Types of Mild Cognitive Impairment. J Med Syst. 2020;44: 107.

19. Gupta U, Bansal H, Joshi D. An improved sex-specific and age-dependent classification model for Parkinson's diagnosis using handwriting measurement. Comput Methods Programs Biomed. 2020;189: 105305.

20. Iakovakis D, Chaudhuri KR, Klingelhoefer L, Bostantjopoulou S, Katsarou Z, Trivedi D, et al. Screening of Parkinsonian subtle fine-motor impairment from touchscreen typing via deep learning. Sci Rep. 2020;10: 12623.

21. Chen OY, Lipsmeier F, Phan H, Prince J, Taylor KI, Gossens C, et al. Building a Machine-Learning Framework to Remotely Assess Parkinson's Disease Using Smartphones. IEEE Trans Biomed Eng. 2020;67: 3491–3500.

22. Xu S, Pan Z. A novel ensemble of random forest for assisting diagnosis of Parkinson's disease on small handwritten dynamics dataset. Int J Med Inform. 2020;144: 104283.

23. Sajal MSR, Ehsan MT, Vaidyanathan R, Wang S, Aziz T, Mamun KAA. Telemonitoring Parkinson's disease using machine learning by combining tremor and voice analysis. Brain Inform. 2020;7: 12.

24. Rovini E, Maremmani C, Cavallo F. A Wearable System to Objectify Assessment of Motor Tasks for Supporting Parkinson's Disease Diagnosis. Sensors . 2020;20. doi:10.3390/s20092630

25. Karabayir I, Goldman SM, Pappu S, Akbilgic O. Gradient boosting for Parkinson's disease diagnosis from voice recordings. BMC Med Inform Decis Mak. 2020;20: 228.

26. Nair P, Trisno R, Baghini MS, Pendharkar G, Chung H. Predicting Early Stage Drug Induced Parkinsonism using Unsupervised and Supervised Machine Learning. Conf Proc

IEEE Eng Med Biol Soc. 2020;2020: 776–779.

27. Byeon H. Is the Random Forest Algorithm Suitable for Predicting Parkinson’s Disease with Mild Cognitive Impairment out of Parkinson's Disease with Normal Cognition? Int J Environ Res Public Health. 2020;17. doi:10.3390/ijerph17072594

28. Vacchi E, Burrello J, Di Silvestre D, Burrello A, Bolis S, Mauri P, et al. Immune profiling of plasma-derived extracellular vesicles identifies Parkinson disease. Neurol Neuroimmunol Neuroinflamm. 2020;7. doi:10.1212/NXI.0000000000000866

29. Salmanpour MR, Shamsaei M, Rahmim A. Feature selection and machine learning methods for optimal identification and prediction of subtypes in Parkinson’s disease. Comput Methods Programs Biomed. 2021;206: 106131.

30. Classification of PPMI MRI scans with voxel-based morphometry and machine learning to assist in the diagnosis of Parkinson’s disease. Comput Methods Programs Biomed. 2021;198: 105793.

31. Byeon H. Application of Machine Learning Technique to Distinguish Parkinson’s Disease Dementia and Alzheimer's Dementia: Predictive Power of Parkinson's Disease-Related Non-Motor Symptoms and Neuropsychological Profile. J Pers Med. 2020;10. doi:10.3390/jpm10020031

32. Juutinen M, Wang C, Zhu J, Haladjian J, Ruokolainen J, Puustinen J, et al. Parkinson’s disease detection from 20-step walking tests using inertial sensors of a smartphone: Machine learning approach based on an observational case-control study. PLoS One. 2020;15: e0236258.

33. Shiiba T, Arimura Y, Nagano M, Takahashi T, Takaki A. Improvement of classification performance of Parkinson’s disease using shape features for machine learning on dopamine transporter single photon emission computed tomography. PLoS One. 2020;15: e0228289.

34. Bhattacharya D, Sinha N, Prasad S, Pal PK, Saini J, Mangalore S. A New Statistical Framework for Corpus Callosum Sub-Region Characterization Based on LBP Texture in Patients With Parkinsonian Disorders: A Pilot Study. Front Neurosci. 2020;14: 477.

35. Chen Y, Zhu G, Liu D, Liu Y, Yuan T, Zhang X, et al. The morphology of thalamic subnuclei in Parkinson’s disease and the effects of machine learning on disease diagnosis and clinical evaluation. J Neurol Sci. 2020;411: 116721.

36. Akbilgic O, Kamaleswaran R, Mohammed A, Ross GW, Masaki K, Petrovitch H, et al. Electrocardiographic changes predate Parkinson’s disease onset. Sci Rep. 2020;10: 11319.

37. Nawar A, Rahman F, Krishnamurthi N, Som A, Turaga P. Topological Descriptors for Parkinson’s Disease Classification and Regression Analysis. Conf Proc IEEE Eng Med Biol Soc. 2020;2020: 793–797.

38. Salmanpour MR, Shamsaei M, Saberi A, Hajianfar G, Soltanian-Zadeh H, Rahmim A. Robust identification of Parkinson’s disease subtypes using radiomics and hybrid machine learning. Comput Biol Med. 2021;129: 104142.

39. Habets JGV, Janssen MLF, Duits AA, Sijben LCJ, Mulders AEP, De Greef B, et al. Machine learning prediction of motor response after deep brain stimulation in Parkinson's disease-proof of principle in a retrospective cohort. PeerJ. 2020;8: e10317.

40. Byeon H. Best early-onset Parkinson dementia predictor using ensemble learning among Parkinson's symptoms, rapid eye movement sleep disorder, and neuropsychological profile. World J Psychiatry. 2020;10: 245–259.

41. Hsu S-Y, Yeh L-R, Chen T-B, Du W-C, Huang Y-H, Twan W-H, et al. Classification of the Multiple Stages of Parkinson's Disease by a Deep Convolution Neural Network Based on Tc-TRODAT-1 SPECT Images. Molecules. 2020;25. doi:10.3390/molecules25204792

42. Yang Y, Wei L, Hu Y, Wu Y, Hu L, Nie S. Classification of Parkinson's disease based on multi-modal features and stacking ensemble learning. J Neurosci Methods. 2021;350: 109019.

43. Williams S, Relton SD, Fang H, Alty J, Qahwaji R, Graham CD, et al. Supervised classification of bradykinesia in Parkinson's disease from smartphone videos. Artif Intell Med. 2020;110: 101966.

44. Rehman RZU, Klocke P, Hryniv S, Galna B, Rochester L, Del Din S, et al. Turning Detection During Gait: Algorithm Validation and Influence of Sensor Location and Turning Characteristics in the Classification of Parkinson's Disease. Sensors . 2020;20. doi:10.3390/s20185377

45. Ghaderyan P, Ghoreshi Beyrami SM. Neurodegenerative diseases detection using distance metrics and sparse coding: A new perspective on gait symmetric features. Comput Biol Med. 2020;120: 103736.

46. Polat K, Nour M. Parkinson disease classification using one against all based data sampling with the acoustic features from the speech signals. Med Hypotheses. 2020;140: 109678.

47. Avuçlu E, Elen A. Evaluation of train and test performance of machine learning algorithms and Parkinson diagnosis with statistical measurements. Med Biol Eng Comput. 2020;58: 2775–2788.

48. Pietrucci D, Teofani A, Unida V, Cerroni R, Biocca S, Stefani A, et al. Can Gut Microbiota Be a Good Predictor for Parkinson's Disease? A Machine Learning Approach. Brain Sci. 2020;10. doi:10.3390/brainsci10040242

49. Virreira Winter S, Karayel O, Strauss MT, Padmanabhan S, Surface M, Merchant K, et al. Urinary proteome profiling for stratifying patients with familial Parkinson's disease. EMBO Mol Med. 2021;13: e13257.

50. Li Y, Zhang S, Odeh C. Automated Classification of Postural Control for Individuals With Parkinson's Disease Using a Machine Learning Approach: A Preliminary Study. J Appl Biomech. 2020; 1–6.

51. Chen Y, Zhu G, Liu D, Liu Y, Yuan T, Zhang X, et al. Brain morphological changes in hypokinetic dysarthria of Parkinson's disease and use of machine learning to predict severity. CNS Neurosci Ther. 2020;26: 711–719.

52. Veeraragavan S, Gopalai AA, Gouwanda D, Ahmad SA. Parkinson's Disease Diagnosis and Severity Assessment Using Ground Reaction Forces and Neural Networks. Frontiers in Physiology. 2020. doi:10.3389/fphys.2020.587057

53. Jin B, Qu Y, Zhang L, Gao Z. Diagnosing Parkinson Disease Through Facial Expression Recognition: Video Analysis. J Med Internet Res. 2020;22: e18697.

54. Pang Y, Christenson J, Jiang F, Lei T, Rhoades R, Kern D, et al. Automatic detection and quantification of hand movements toward development of an objective assessment of tremor and bradykinesia in Parkinson's disease. J Neurosci Methods. 2020;333: 108576.

55. Peralta M, Baxter JSH, Khan AR, Haegelen C, Jannin P. Striatal shape alteration as a staging biomarker for Parkinson's Disease. Neuroimage Clin. 2020;27: 102272.

56. Correia MM, Rittman T, Barnes CL, Coyle-Gilchrist IT, Ghosh B, Hughes LE, et al. Towards accurate and unbiased imaging-based differentiation of Parkinson's disease, progressive supranuclear palsy and corticobasal syndrome. Brain Commun. 2020;2: fcaa051.

57. Zhang A, De la Torre F, Hodgins J. Comparing laboratory and in-the-wild data for continuous Parkinson's Disease tremor detection. Conf Proc IEEE Eng Med Biol Soc. 2020;2020: 5436–5441.

58. Prediction and Estimation of Parkinson's Disease Severity Based on Voice Signal. J Voice. 2022;36: 439.e9–439.e20.

59. de Araújo ACA, Santos EG da R, de Sá KSG, Furtado VKT, Santos FA, de Lima RC, et al. Hand Resting Tremor Assessment of Healthy and Patients With Parkinson's Disease: An Exploratory Machine Learning Study. Front Bioeng Biotechnol. 2020;8: 778.

60. Yaman O, Ertam F, Tuncer T. Automated Parkinson's disease recognition based on statistical pooling method using acoustic features. Med Hypotheses. 2020;135: 109483.

61. Zhang XB, Zhai DH, Yang Y, Zhang YL, Wang CL. A novel semi-supervised multi-view clustering framework for screening Parkinson's disease. Math Biosci Eng. 2020;17: 3395–3411.

62. Emon MA, Heinson A, Wu P, Domingo-Fernández D, Sood M, Vrooman H, et al. Clustering of Alzheimer's and Parkinson's disease based on genetic burden of shared molecular mechanisms. Sci Rep. 2020;10: 19097.

63. Parziale A, Senatore R, Della Cioppa A, Marcelli A. Cartesian genetic programming for diagnosis of Parkinson disease through handwriting analysis: Performance vs. interpretability issues. Artif Intell Med. 2021;111: 101984.

64. Butt AH, Cavallo F, Maremmani C, Rovini E. Biomechanical parameters assessment for the classification of Parkinson Disease using Bidirectional Long Short-Term Memory. Conf Proc IEEE Eng Med Biol Soc. 2020;2020: 5761–5764.

65. Chang Z, Chen Z, Stephen CD, Schmahmann JD, Wu H-T, Sapiro G, et al. Accurate detection of cerebellar smooth pursuit eye movement abnormalities via mobile phone video and machine learning. Sci Rep. 2020;10: 18641.

66. Tracy JM, Özkanca Y, Atkins DC, Hosseini Ghomi R. Investigating voice as a biomarker:

Deep phenotyping methods for early detection of Parkinson's disease. J Biomed Inform. 2020;104: 103362.

67. Papadopoulos A, Iakovakis D, Klingelhoefer L, Bostantjopoulou S, Chaudhuri KR, Kyritsis K, et al. Unobtrusive detection of Parkinson's disease from multi-modal and in-the-wild sensor data using deep learning techniques. Sci Rep. 2020;10: 21370.

68. Xu S, Wang Z, Sun J, Zhang Z, Wu Z, Yang T, et al. Using a deep recurrent neural network with EEG signal to detect Parkinson's disease. Ann Transl Med. 2020;8: 874.

69. Chakraborty S, Aich S, Kim H-C. 3D Textural, Morphological and Statistical Analysis of Voxel of Interests in 3T MRI Scans for the Detection of Parkinson's Disease Using Artificial Neural Networks. Healthcare (Basel). 2020;8. doi:10.3390/healthcare8010034

70. Varghese J, Fujarski M, Hahn T, Dugas M, Warnecke T. The Smart Device System for Movement Disorders: Preliminary Evaluation of Diagnostic Accuracy in a Prospective Study. Stud Health Technol Inform. 2020;270: 889–893.

71. Tsai C-C, Lin Y-C, Ng S-H, Chen Y-L, Cheng J-S, Lu C-S, et al. A Method for the Prediction of Clinical Outcome Using Diffusion Magnetic Resonance Imaging: Application on Parkinson's Disease. J Clin Med Res. 2020;9. doi:10.3390/jcm9030647

72. Tougui I, Jilbab A, Mhamdi JE. Analysis of Smartphone Recordings in Time, Frequency, and Cepstral Domains to Classify Parkinson's Disease. Healthc Inform Res. 2020;26: 274–283.

73. Falchetti M, Prediger RD, Zanotto-Filho A. Classification algorithms applied to blood-based transcriptome meta-analysis to predict idiopathic Parkinson's disease. Comput Biol Med. 2020;124: 103925.

74. Chakraborty S, Aich S, Kim H-C. Detection of Parkinson's Disease from 3T T1 Weighted MRI Scans Using 3D Convolutional Neural Network. Diagnostics (Basel). 2020;10. doi:10.3390/diagnostics10060402

75. Aouraghe I, Alae A, Ghizlane K, Mrabti M, Aboulem G, Faouzi B. A novel approach combining temporal and spectral features of Arabic online handwriting for Parkinson's disease prediction. J Neurosci Methods. 2020;339: 108727.

76. Leger C, Herbert M, DeSouza JFX. Non-motor Clinical and Biomarker Predictors Enable High Cross-Validated Accuracy Detection of Early PD but Lesser Cross-Validated Accuracy Detection of Scans Without Evidence of Dopaminergic Deficit. Front Neurol. 2020;11: 364.

77. Tremblay C, Mei J, Frasnelli J. Olfactory bulb surroundings can help to distinguish Parkinson's disease from non-parkinsonian olfactory dysfunction. Neuroimage Clin. 2020;28: 102457.

78. Lin C-W, Wen T-C, Setiawan F. Evaluation of Vertical Ground Reaction Forces Pattern Visualization in Neurodegenerative Diseases Identification Using Deep Learning and Recurrence Plot Image Feature Extraction. Sensors . 2020;20. doi:10.3390/s20143857

79. Eyigoz E, Courson M, Sedeño L, Rogg K, Orozco-Arroyave JR, Nöth E, et al. From discourse to pathology: Automatic identification of Parkinson's disease patients via morphological measures across three languages. Cortex. 2020;132: 191–205.

80. Som A, Krishnamurthi N, Buman M, Turaga P. Unsupervised Pre-trained Models from Healthy ADLs Improve Parkinson's Disease Classification of Gait Patterns. Conf Proc IEEE Eng Med Biol Soc. 2020;2020: 784–788.

81. Tunc HC, Sakar CO, Apaydin H, Serbes G, Gunduz A, Tutuncu M, et al. Estimation of Parkinson's disease severity using speech features and extreme gradient boosting. Med Biol Eng Comput. 2020;58: 2757–2773.

82. Di Lazzaro G, Ricci M, Al-Wardat M, Schirinzi T, Scalise S, Giannini F, et al. Technology-Based Objective Measures Detect Subclinical Axial Signs in Untreated, de novo Parkinson's Disease. J Parkinsons Dis. 2020;10: 113–122.

83. Singh S, Xu W. Robust Detection of Parkinson's Disease Using Harvested Smartphone Voice Data: A Telemedicine Approach. Telemed J E Health. 2020;26: 327–334.

84. Shahid AH, Singh MP. A deep learning approach for prediction of Parkinson's disease progression. Biomed Eng Lett. 2020;10: 227–239.

85. Mohammed F, He X, Lin Y. An easy-to-use deep-learning model for highly accurate diagnosis of Parkinson's disease using SPECT images. Comput Med Imaging Graph. 2021;87: 101810.

86. Bougea A, Efthymiopoulou E, Spanou I, Zikos P. A Novel Machine Learning Algorithm Predicts Dementia With Lewy Bodies Versus Parkinson's Disease Dementia Based on Clinical and Neuropsychological Scores. J Geriatr Psychiatry Neurol. 2022;35: 317–320.

87. Talai AS, Sedlacik J, Boelmans K, Forkert ND. Utility of Multi-Modal MRI for Differentiating of Parkinson's Disease and Progressive Supranuclear Palsy Using Machine Learning. Front Neurol. 2021;12: 648548.

88. Huang Z, Lei H, Chen G, Frangi AF, Xu Y, Elazab A, et al. Parkinson's Disease Classification and Clinical Score Regression via United Embedding and Sparse Learning From Longitudinal Data. IEEE Trans Neural Netw Learn Syst. 2022;33: 3357–3371.

89. Martins R, Oliveira F, Moreira F, Moreira AP, Abrunhosa A, Januário C, et al. Automatic classification of idiopathic Parkinson's disease and atypical Parkinsonian syndromes combining [C]raclopride PET uptake and MRI grey matter morphometry. J Neural Eng. 2021;18. doi:10.1088/1741-2552/abf772

90. Zhang X, Cao X, Xue C, Zheng J, Zhang S, Huang Q, et al. Aberrant functional connectivity and activity in Parkinson's disease and comorbidity with depression based on radiomic analysis. Brain Behav. 2021;11: e02103.

91. Yasaka K, Kamagata K, Ogawa T, Hatano T, Takeshige-Amano H, Ogaki K, et al. Parkinson's disease: deep learning with a parameter-weighted structural connectome matrix for diagnosis and neural circuit disorder investigation. Neuroradiology. 2021;63: 1451–1462.

92. Byeon H. Predicting the Severity of Parkinson's Disease Dementia by Assessing the Neuropsychiatric Symptoms with an SVM Regression Model. Int J Environ Res Public Health. 2021;18. doi:10.3390/ijerph18052551

93. de Souza RWR, Silva DS, Passos LA, Roder M, Santana MC, Pinheiro PR, et al. Computer-assisted Parkinson’s disease diagnosis using fuzzy optimum- path forest and Restricted Boltzmann Machines. Comput Biol Med. 2021;131: 104260.

94. Vu JP, Yamin G, Reyes Z, Shin A, Young A, Litvan I, et al. Assessment of Motor Dysfunction with Virtual Reality in Patients Undergoing [I]FP-CIT SPECT/CT Brain Imaging. Tomography. 2021;7: 95–106.

95. Suo X, Lei D, Li N, Li W, Kemp GJ, Sweeney JA, et al. Disrupted morphological grey matter networks in early-stage Parkinson’s disease. Brain Struct Funct. 2021;226: 1389–1403.

96. Mangesius S, Mariotto S, Ferrari S, Pereverzyev S Jr, Lerchner H, Haider L, et al. Novel decision algorithm to discriminate parkinsonism with combined blood and imaging biomarkers. Parkinsonism Relat Disord. 2020;77: 57–63.

97. Peng X, Feng Y, Ji S, Amos JT, Wang W, Li M, et al. Gait Analysis by Causal Decomposition. IEEE Trans Neural Syst Rehabil Eng. 2021;29: 953–964.

98. Jha A, Menozzi E, Oyekan R, Latorre A, Mulroy E, Schreglmann SR, et al. The CloudUPDRS smartphone software in Parkinson’s study: cross-validation against blinded human raters. NPJ Parkinsons Dis. 2020;6: 36.

99. Shahtalebi S, Atashzar SF, Patel RV, Jog MS, Mohammadi A. A deep explainable artificial intelligent framework for neurological disorders discrimination. Sci Rep. 2021;11: 9630.

100. Nguyen AA, Maia PD, Gao X, F Damasceno P, Raj A. Dynamical Role of Pivotal Brain Regions in Parkinson Symptomatology Uncovered with Deep Learning. Brain Sci. 2020;10. doi:10.3390/brainsci10020073

101. Phongpreecha T, Cholerton B, Mata IF, Zabetian CP, Aghaeepour N, Tian L, et al. Multivariate prediction of dementia in Parkinson’s disease. npj Parkinson’s Disease. 2020;6: 1–10.

102. Peres LB, Calil BC, da Silva APSPB, Dionísio VC, Vieira MF, de Oliveira Andrade A, et al. Discrimination between healthy and patients with Parkinson’s disease from hand resting activity using inertial measurement unit. Biomed Eng Online. 2021;20: 50.

103. Sieberts SK, Schaff J, Duda M, Pataki BÁ, Sun M, Snyder P, et al. Crowdsourcing digital health measures to predict Parkinson’s disease severity: the Parkinson's Disease Digital Biomarker DREAM Challenge. NPJ Digit Med. 2021;4: 53.

104. Sabo A, Mehdizadeh S, Ng K-D, Iaboni A, Taati B. Assessment of Parkinsonian gait in older adults with dementia via human pose tracking in video data. J Neuroeng Rehabil. 2020;17: 97.

105. E B, D B, Elumalai VK, K U. Data-driven gait analysis for diagnosis and severity rating of Parkinson’s disease. Med Eng Phys. 2021;91: 54–64.

106. Williamson JR, Telfer B, Mullany R, Friedl KE. Detecting Parkinson’s Disease from Wrist-Worn Accelerometry in the U.K. Biobank. Sensors . 2021;21. doi:10.3390/s21062047

107. Nguyen KP, Raval V, Treacher A, Mellema C, Yu FF, Pinho MC, et al. Predicting Parkinson’s disease trajectory using clinical and neuroimaging baseline measures.

Parkinsonism Relat Disord. 2021;85: 44–51.

108. Shi D, Zhang H, Wang S, Wang G, Ren K. Application of Functional Magnetic Resonance Imaging in the Diagnosis of Parkinson’s Disease: A Histogram Analysis. Front Aging Neurosci. 2021;13: 624731.

109. Ma L-Y, Tian Y, Pan C-R, Chen Z-L, Ling Y, Ren K, et al. Motor Progression in Early-Stage Parkinson’s Disease: A Clinical Prediction Model and the Role of Cerebrospinal Fluid Biomarkers. Front Aging Neurosci. 2020;12: 627199.

110. Jeancolas L, Petrovska-Delacrétaz D, Mangone G, Benkelfat B-E, Corvol J-C, Vidailhet M, et al. X-Vectors: New Quantitative Biomarkers for Early Parkinson’s Disease Detection From Speech. Front Neuroinform. 2021;15: 578369.

111. Vacchi E, Burrello J, Burrello A, Bolis S, Monticone S, Barile L, et al. Profiling Inflammatory Extracellular Vesicles in Plasma and Cerebrospinal Fluid: An Optimized Diagnostic Model for Parkinson’s Disease. Biomedicines. 2021;9. doi:10.3390/biomedicines9030230

112. Adams MP, Rahmim A, Tang J. Improved motor outcome prediction in Parkinson’s disease applying deep learning to DaTscan SPECT images. Comput Biol Med. 2021;132: 104312.

113. Dai H, Cai G, Lin Z, Wang Z, Ye Q. Validation of Inertial Sensing-Based Wearable Device for Tremor and Bradykinesia Quantification. IEEE J Biomed Health Inform. 2021;25: 997–1005.
