# Supplementary material for: Machine Learning Models for Parkinson Disease: Systematic Review
Source: JMIR Med Inform. 2024 May 17;12:e50117. doi: 10.2196/50117 (PMC11112052; doi:10.2196/50117)
Supplement: Multimedia Appendix 3 [file medinform-v12-e50117-s003.docx]

Multimedia Appendix 3. Graphical representations of data.

**Figure S1.** Distribution of studies according to data set splitting techniques.

**Figure S2.** Distribution of studies that adopted cross-validation techniques; More than half of the studies applied k-fold cross-validation.

**Figure S3.** Percentage of studies according to hyperparameter tuning method; over one-third of studies did not report on hyperparameter tuning.

**Figure S4.** Model comparison methods; 18.6% of studies did not compare their model results to any alternative models or previously published models/benchmarks.
